# A comparison of endolymphatic duct blockage, endolymphatic sac drainage and endolymphatic sac decompression surgery in reversing endolymphatic hydrops in Meniere’s disease

**DOI:** 10.1186/s40463-021-00545-7

**Published:** 2021-12-20

**Authors:** Anquan Peng, Junjiao Hu, Qin Wang, Xueying Pan, Zhiwen Zhang, Wenqi Jiang, Yichao Chen, Chao Huang

**Affiliations:** 1 Department of Otolaryngology-Head and Neck Surgery, The Second Xiangya Hospital, Central South University, Changsha, 410011 Hunan China; 2 Department of Radiology, The Second Xiangya Hospital, Central South University, 139 Middle Renmin Road, Changsha, 410011 Hunan China

**Keywords:** Meniere’s disease, Endolymphatic duct blockage, Endolymphatic sac drainage, Endolymphatic hydrops

## Abstract

**Background:**

To explore the differences between endolymphatic duct blockage, endolymphatic sac drainage and endolymphatic sac decompression surgery in the reversal of endolymphatic hydrops (EH) in patients with intractable Meniere’s disease (MD).

**Methods:**

A total of 27 MD patients receiving endolymphatic duct blockage surgery (n = 10), endolymphatic sac drainage surgery (n = 9) and endolymphatic sac decompression surgery (n = 8) underwent gadolinium-enhanced inner ear magnetic resonance imaging (MRI) scans prior to, 2 weeks after and at > 12 months following surgery.

**Results:**

In the group with endolymphatic duct blockage, the second MRI revealed no changes in EH, whereas the third MRI revealed a reversal of vestibular EH in 3 patients and a downgrading of cochlear hydrops in 2 of these 3 patients, who presented with an improvement in their hearing and complete control of vertigo. In the group with endolymphatic sac drainage, the second MRI showed a reversal of EH in 4 patients, and no changes in EH in the remaining 5 patients, whereas the third MRI showed that those 4 patients who presented with a reversal of EH at the second MRI stage remained unchanged except a recurrence of vestibular hydrops in 1 patient. All 4 patients exhibited a complete control of vertigo, but hearing improved in 1, worsened in 1 and remained unchanged in 2. In the group with endolymphatic sac decompression, both the second and third MRI examination revealed no reversal of EH.

**Conclusions:**

The present study has shown that both endolymphatic duct blockage surgery and endolymphatic sac drainage surgery have the potential to reduce EH in certain MD patients, but none of the patients receiving endolymphatic sac decompression surgery showed reversal of their EH.

**Graphical Abstract:**

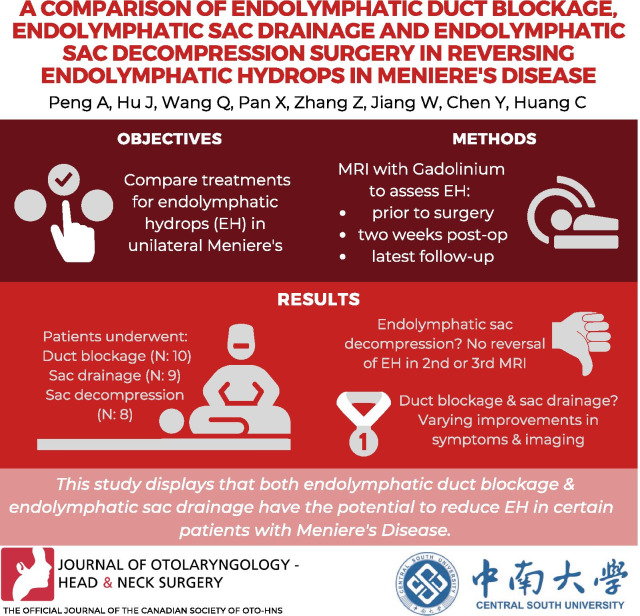

## Introduction

Meniere’s disease (MD) is characterized by episodic vertigo, fluctuating sensorineural hearing loss and aural symptoms [1]. Although the specific underlying pathophysiological mechanisms of MD remain unknown, the presumed cause of the symptoms experienced in MD is considered to be due to endolymphatic hydrops (EH), which is characterized by an enlargement of the cochlear and vestibular endolymphatic space [1]. EH is considered to be caused by immune, metabolic, infectious, traumatic, or other insults to the inner ear associated with a temporarily dysfunctional endolymphatic sac [2, 3]. Consequently, when conventional medical treatments for MD fail, endolymphatic sac surgery, as the first surgical procedure, is usually performed according to the treatment guidelines for MD [4]. The effectiveness of endolymphatic sac drainage surgery, which was described by Portmann in 1927, was considered to be achieved through opening the endolymphatic sac, reducing the endolymphatic pressure for MD [5]. whereas endolymphatic sac decompression is speculated to improve the absorption of endolymph in sac [6]. More recently, a novel surgical sac technique for the treatment of MD, endolymphatic duct blockage, was shown be effective for the control of symptoms of MD, without any noticeable cochlear and vestibular damage [7, 8]. The procedure was also considered to decrease hydrops, most likely due to a reduction in the volume of the endolymph in the inner ear coming from the sac. However, the efficacy of these treatments has not been proved, and the mechanisms underlying their effects remain speculative. With such sac surgery, it is unclear whether the EH in patients with MD can be reduced, and if it should happen, it is not clear when the reduction in EH occurs or how the reduction of EH is associated with the dynamics of hearing threshold and vertigo attacks.

To improve our understanding of these matters, in the present study, the dynamics of EH were evaluated using a gadolinium (Gd)-enhanced inner ear magnetic resonance imaging (MRI) technique at three time points: (i) prior to surgery; (ii) 2 weeks after surgery; and (iii) at the latest follow-up, with a > 12-month interval after surgery in patients with intractable MD who underwent endolymphatic duct blockage, endolymphatic sac drainage or endolymphatic sac decompression surgery. The aim of the present study was to determine the comparative effectiveness of the three techniques and the different time points in terms of reversing EH in patients with MD.

## Methods

### Patients

The diagnostic criteria for MD jointly formulated by the Classification Committee of the Bárány Society [4] were closely followed. Twenty-six patients with unilateral definite MD and MRI-based visualization of unilateral EH underwent endolymphatic duct blockage surgery, 18 patients with unilateral definite MD and MRI-based visualization of unilateral EH underwent endolymphatic sac drainage surgery and 12 patients with unilateral definite MD and MRI-based visualization of unilateral EH underwent endolymphatic sac decompression surgery for treatment of intractable MD at our University Hospital between June 2017 and November 2019. In all the patients enrolled in the present study, vestibular migraine has been ruled out using the Bárány diagnostic criteria [4]. Intractable MD in patients was defined as recurrent vertigo/dizziness for ≥ 6 months with a failure of systematic medical treatment, including the administration of osmotic diuretic medicine and betahistine, psychological management and use of intratympanic steroids or gentamycin.

All the participants were instructed to discontinue all other treatments for MD and keep a daily vertigo diary to document the occurrence of MD attacks following surgery. Definitive vertigo/dizziness lasting > 20 min was considered as a vertigo attack [4]. Based on a suggestion by Gȕrkov et al. [9], the frequency of vertigo spells before surgery was calculated based on the number of vertigo attacks during the 6 months prior to surgery. Frequency of vertigo spells following surgery was calculated based on the number of vertigo attacks during the latest 6 months following surgery. A numerical value for reporting vertigo control was calculated using the following formula [10]: Numerical value (NV) = (X/Y) × 100, rounded to the nearest whole number, where X is the average number of definitive spells per month for the latest 6 months following surgery and Y is the average number of definitive spells per month for the 6 months prior to surgery. According to AAO-HNS vertigo control index [10], control of vertigo was classified as complete control (NV = 0), substantial control (NV = 1–40), limited control (NV = 41–80), insignificant control (NV = 81–120) and worse control of vertigo (NV > 120).

Hearing level was measured using a pure-tone audiometer (SM950; Inmedico A/S, Denmark) and was evaluated based on the mean values calculated from air-conduction hearing threshold levels at 500, 1000, 2000, and 3000 Hz. The hearing level before surgery was determined by evaluating the worst hearing level during the 6 months prior to surgery, whereas the hearing level following surgery was determined by evaluating the worst hearing level during the most recent 6 months following surgery. According to the average hearing threshold in individuals, changes in hearing levels were defined: Worse, elevation ≥ 10 dB; Better, Decline ≥ 10 dB; And same, if no more than 10 dB changes.

The present study was approved by the Medical Ethics Committee of the Second Xiangya Hospital (certificate number: S452). All participants provided their written informed consent in accordance with the Declaration of Helsinki. All patients who were followed-up for Gd-MRI were fully informed about the execution and goals of the study and provided informed consent to participate in this study for evaluating the dynamics of their EH.

### Gd-MRI administration

MRI was performed as previously described [11, 12]. In brief, when a single-dose (0.2 ml/kg) gadolinium-based contrast agent (Magnevist®, Bayer AG) was administrated intravenously 4 h before the MRI scan (IV-Gd) and a eightfold-diluted Gd was injected intratympanically in both ears 24 h prior to the MRI scan (IT-Gd), the MRI scan was performed with a three-dimensional real inversion recovery (3D-real IR) sequence on a 3 T MR unit (Magnetom Verio; Siemens AG) using a 12-channel head coil, as previously described [11, 12]. For the second and third Gd-MRI, the intratympanic administration of contrast agent might be used in the ear undergoing surgery alone when patients were reluctant to receive intratympanic injections in their healthy ear. Therefore, all the surgical ears received IT-Gd + IV-Gd MRI using essentially the same imaging technique in the first, second and third Gd-MRI. The off-label use of IT-Gd-MRI was performed under informed consent.

### Image evaluation

**Fig. 1:**
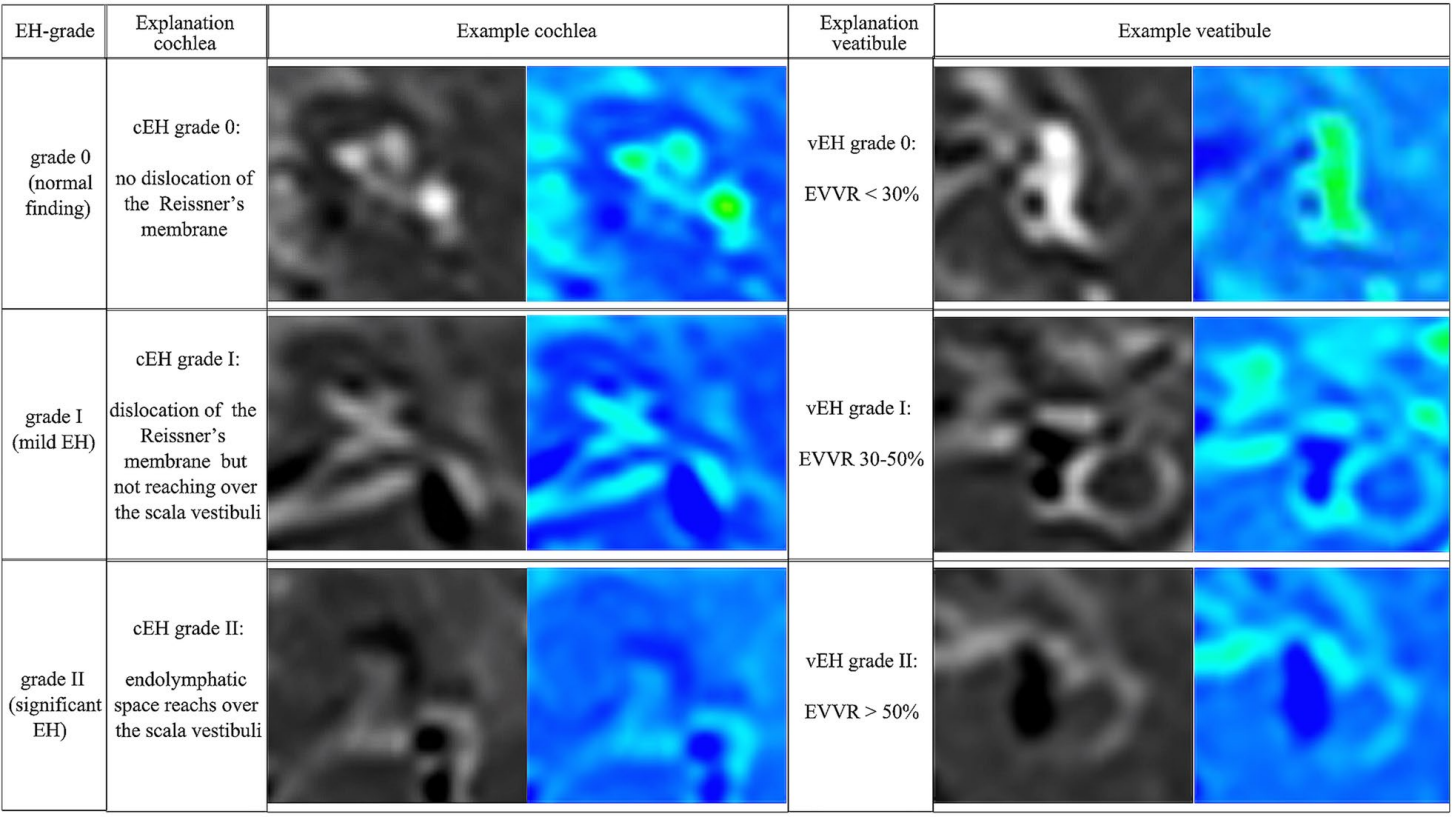
Criteria for grading of the cochlear and vestibular hydrops in three-dimensional fluid-attenuated inversion recovery MRI axial scans in the left column, and the corresponding MRI colored image in the right column. MRI, magnetic resonance imaging

The MR image analysis was performed by two experienced head and neck radiologists who were blinded to the treatment of all patients. The corresponding MRI colored image could be achieved by the pseudocolor technique, where the blue color represents the endolymphatic spaces and the green color represents the perilymphatic spaces in the cochlea and vestibule. The degree of EH was determined as previously described [13]. In brief, the cochlear hydrops was classified as none (normal finding without EH), grade I (mild EH) or grade II (significant EH) (Fig. 1A). Based on the volume-ratio of endolymphatic space to the total vestibule (endolymph to vestibule-volume ratio, or EVVR), the vestibular EH was classified as follows: None (or grade N), < 30% of the vestibular space was filled with endolymph; grade I, 30–50% of the vestibular space was filled with endolymph; and grade II, > 50% of the vestibular space was filled with endolymph (Fig. 1B). Change in the vestibular hydrops was defined as previously described by Uno et al. [14]. An EVVR increase of > 10 percentage (%) was defined as an increase of vestibular hydrops, whereas an EVVR decrease of > 10 percentage (%) was defined as a reduction of vestibular hydrops. An EVVR change of ≤ 10 percentage (%) was defined as no change in the vestibular hydrops, and an EVVR of < 30 percentage (%) was defined as the complete reversal of vestibular hydrops.

### Surgical techniques

The surgical procedure used for endolymphatic sac decompression was similar to that previously described by Durland Jr et al. [6]. Briefly, a simple mastoidectomy was performed to expose the endolymphatic sac in the area between the sigmoid sinus and the inferior margin of the posterior semicircular canal, including the rugose portion. The surgical procedure used for endolymphatic duct blackage was performed as previously described [7, 12, 13]. Briefly, following decompression of the sac, the retrolabyrinthine bone medial to the sac was dissected to create a region for the insertion of the tips of the forceps. A pair of small titanium clips was used to block the dissected endolymphatic duct using the ligating clip applier. By contrast, the surgical procedure used for endolymphatic sac drainage was similar to that previously described by Kitahara et al. [15]. Briefly, following decompression of the sac, the extraosseous endolymphatic sac was opened with an L-shaped incision for drainage of excess endolymphatic fluids in sac.

For the statistical analysis, paired Student’s t-tests was used for the analysis of two groups. P < 0.05 was considered to indicate a statistically significant difference. SPSS (version 26.0; IBM Corp.) software was used for the statistical analysis.

## Results

In order to compare the dynamic changes of EH between the patients enrolled in the present study in three groups, a second MRI examination was performed 2 weeks after surgery, and a subsequent third MRI examination was performed at a > 12-month interval following surgery. There were a total of 10 subjects in the group with endolymphatic duct blackage (5 females and 5 males; age range, 37–62 years; mean age, 49.4 years), 9 subjects in the group with endolymphatic sac drainage (6 females and 3 males; age range, 29–66 years; mean age, 48.1 years) and 8 subjects in the group with endolymphatic sac decompression (5 females and 3 males; age range, 37–73 years; mean age, 49.4 years) who met the inclusion criteria. Table 1 shows the preoperative and postoperative clinical profiles of the 27 patients enrolled in the present study. No statistical difference in age, gender, side, duration of symptoms, follow-up duration, preoperative hearing threshold or number of vertigo attacks was identified between three groups (P > 0.05).

**Table 1:** Clinical profiles of all 27 patients, including case nos. 1–10 in the EDB group (n = 10), case nos. 11–19 in the EDD group (n = 9) and case nos. 20–27 in the ESD group (n = 8)

Case no.	Age/gender	Side	endolymphatic sac surgery	Disease of duration (months)	Hearing (dB)	Vertigo Attacks (a/mon)			EVVR (%)			CH			Follow-up (months)
					Pre	Post	Pre	Post	First	Second	Third	First	Second	Third	
1	37/M	L	EDB	10	53.8.	47.5	4.2	1.8	56.42	58.25	59.55	I	I	I	21
2	39/F	R	EDB	22	**42.5**	** 27.5**	3.8	0	74.68	76.55	**15.45**	I	I	**N**	20
3	61/F	L	EDB	14	57.5	62.5	3.8	0.7	46.55	45.60	52.10	I	I	I	19
4	52/F	L	EDB	16	41.3	57.5	3.0	3.3	37.85	39.80	52.30	I	I	II	18
5	46/M	R	EDB	32	**58.8**	** 38.8**	2.5	0	82.65	84.35	**47.45**	II	II	II	18
6	48/M	R	EDB	16	52.5	56.3	2.5	0	54.55	58.75	46.25	I	I	I	16
7	62/F	R	EDB	66	**48.8**	** 21.3**	4.3	0	78.73	82.45	**13.89**	II	II	**I**	16
8	48/M	L	EDB	12	42.5	45	3.7	1.7	41.30	40.28	45.15	I	I	I	15
9	52/M	L	EDB	42	62.5	60	2.5	0	66.82	68.30	64.25	II	II	II	14
10	49/F	R	EDB	12	45	46.3	3.3	0	50.65	53.58	47.48	I	I	1	13
11	29/F	R	EDD	45	63.8	60	4.2	0.5	78.65	76.45	79.35	II	II	II	26
12	49/M	L	EDD	12	52.5	67.5	3.3	0	72.55	**18.25**	**25.35**	I	**N**	**N**	22
13	48/M	R	EDD	36	57.5	63.8	2.8	1.8	66.45	68.33	78.66	II	II	II	17
14	66/F	L	EDD	55	65	68.8	4.0	0	62.56	**12.25**	65.30	I	**N**	**N**	17
15	39/F	R	EDD	24	**43.8**	** 28.8**	2.5	0	52.66	**15.30**	**18.35**	I	**N**	**N**	15
16	62/F	L	EDD	18	60	57.5	4.3	0	65.60	**10.20**	**18.25**	II	**N**	**N**	15
17	47/F	L	EDD	13	37.5	35	3.8	0	56.44	54.80	52.46	I	I	I	15
18	56/M	R	EDD	72	65	77.5	3.0	0.8	72.22	74.55	76.36	II	II	II	14
19	37/F	L	EDD	11	27.5	33.8	1.8	0.5	48.35	51.25	54.64	I	I	I	13
20	73/F	R	ESD	96	72.5	77.5	1.5	1.2	81.75	83.55	79.45	II	II	II	24
21	40/F	R	ESD	30	57.5	51.3	2.8	0.8	63.30	66.66	64.25	II	II	II	18
22	37/F	R	ESD	24	**47.5**	** 32.5**	1.8	0.5	48.85	49.40	45.36	I	I	I	18
23	60/M	L	ESD	48	65	72.5	1.5	0	72.30	76.35	70.22	II	II	II	15
24	39/M	L	ESD	18	42.5	48.8	3.0	2.3	52.55	54.33	57.28	I	I	I	15
25	49/F	R	ESD	42	52.5	57.5	3.3	0.7	58.25	61.30	63.45	II	II	II	14
26	45/M	L	ESD	11	37.5	48.8	2.5	2.3	45.72	49.35	55.36	I	I	I	14
27	52/F	R	ESD	26	55	51.3	1.8	0	55.65	58.56	59.18	I	I	I	13

The bold number represents a reduction in endolymphatic hydrops or hearing improvement, whereas the underlined number represents an increase in endolymphatic hydrops or hearing worsening prior to and following surgery

a/mon, attacks per month; EVVR, endolymph to vestibule-volume ratio; CH, cochlear hydrops; Pre, pre-surgery; Post, post-surgery; F, female; M, male; R, right; L, left; Mon, month; N, no endolymphatic hydrops; I, mild endolymphatic hydrops; II, significant endolymphatic hydrops; EDB, endolymphatic duct blockage, EDD, endolymphatic sac drainage, ESD, endolymphatic sac decompression

**Fig. 2:**
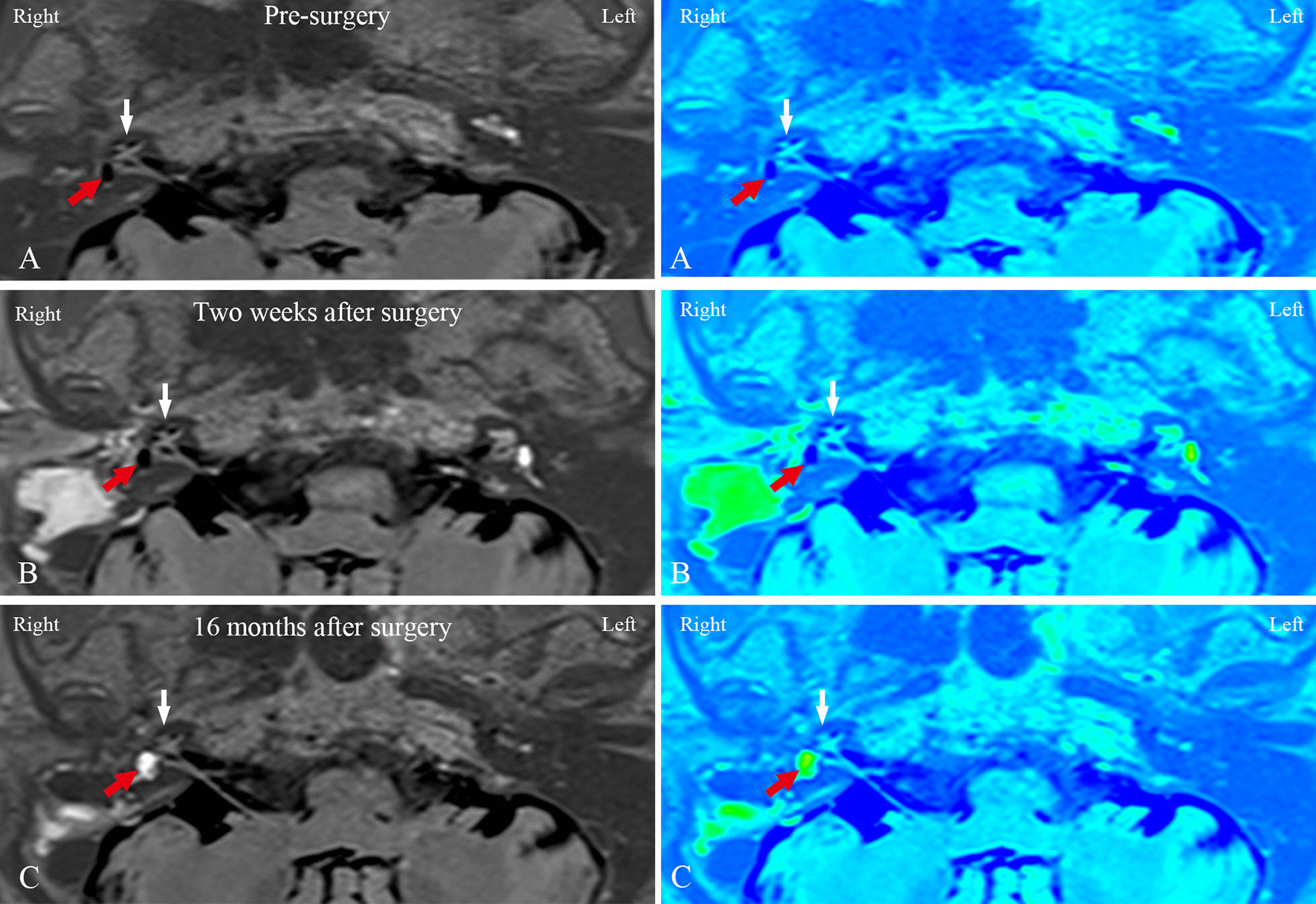
MRI axial scans (left column), and the corresponding MRI colored images (right column) of patient no. 7 (Table 1) in the EDB group with right MD **A** prior to surgery, **B** 2 weeks after surgery, and **C** 16 months after surgery are shown. **A** 3D-real IR MRI revealed a significant EH both in the cochlea (white arrow) and vestibule (red arrow) in the right ear, and no pathological findings were identified in the left ear. **B** Compared with the imaging in the same slice level in the first MRI examination (**A**), the second MRI examination showed that the EH tended to temporarily increase both in the cochlea (white arrow) and the vestibule (red arrow). **C** The third MRI examination showed complete reversal of vestibular hydrops (red arrow) and downgrading of cochlear hydrops (white arrow) from grade II to grade I. *EDB* endolymphatic duct blockage, *EH* endolymphatic hydrops, *MD* Meniere's disease, *3D-real IR MRI* three-dimensional real inversion recovery, *MRI* magnetic resonance imaging

**Fig. 3:**
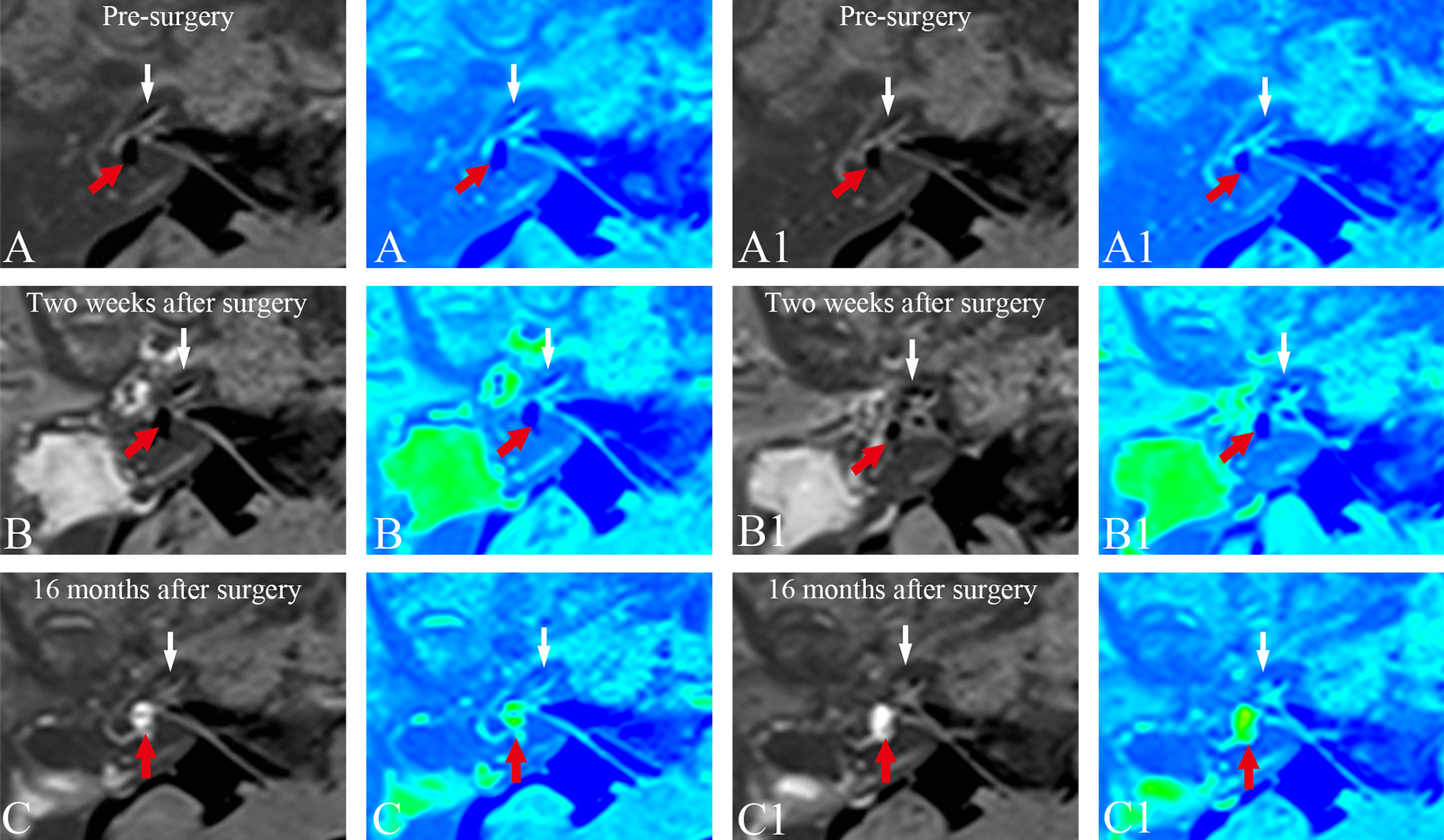
Serial MRI axial scans (left column) and the corresponding MRI colored images (right column) revealed a significant EH both in the cochlea (white arrow) and vestibule (red arrow) prior to surgery (**A, A1**), a tendency for EH to temporarily increase both in the cochlea (white arrow) and the vestibule (red arrow) 2 weeks after surgery (**B, B1**) and a complete reversal of vestibular hydrops (red arrow) and downgrading of cochlear hydrops (white arrow) from grade II to grade I 16 months after surgery (**C, C1**) in the same patient in Fig. 2. *EH* endolymphatic hydrops, *MRI* magnetic resonance imaging

Compared with the pre-surgery recordings (first MRI examination), in the group with endolymphatic duct blackage (patient case nos. 1–10), the second MRI examination revealed no changes in either the vestibular or the cochlear hydrops in any of the subjects. By contrast, the results of the third MRI scans showed a reversal of vestibular EH in 3 patients, accompanied by a downgrading of cochlear hydrops in 2 patients, who also showed an improvement in their hearing and complete control of vertigo attacks. One patient showed an increase in vestibular EH, accompanied by an upgrading of cochlear hydrops and an increase in the number of vertigo attacks. Six patients showed no changes in EH or hearing, and complete control of vertigo attacks in 3 patients, substantial control of vertigo attacks in 1 patient and limited control of vertigo attacks in 2 patients. Figures 2 and 3 show the dynamics of EH in patient no. 7. The second MRI examination revealed that the EVVR increased to 82.45% from 78.73% in the first MRI examination, suggesting that there was a tendency for EH to temporarily increase 2 weeks after surgery, whereas the results of the third MRI showed complete reversal of vestibular hydrops 16 months following surgery, and a concomitant downgrading of cochlear hydrops from grade II to grade I.

**Fig. 4:**
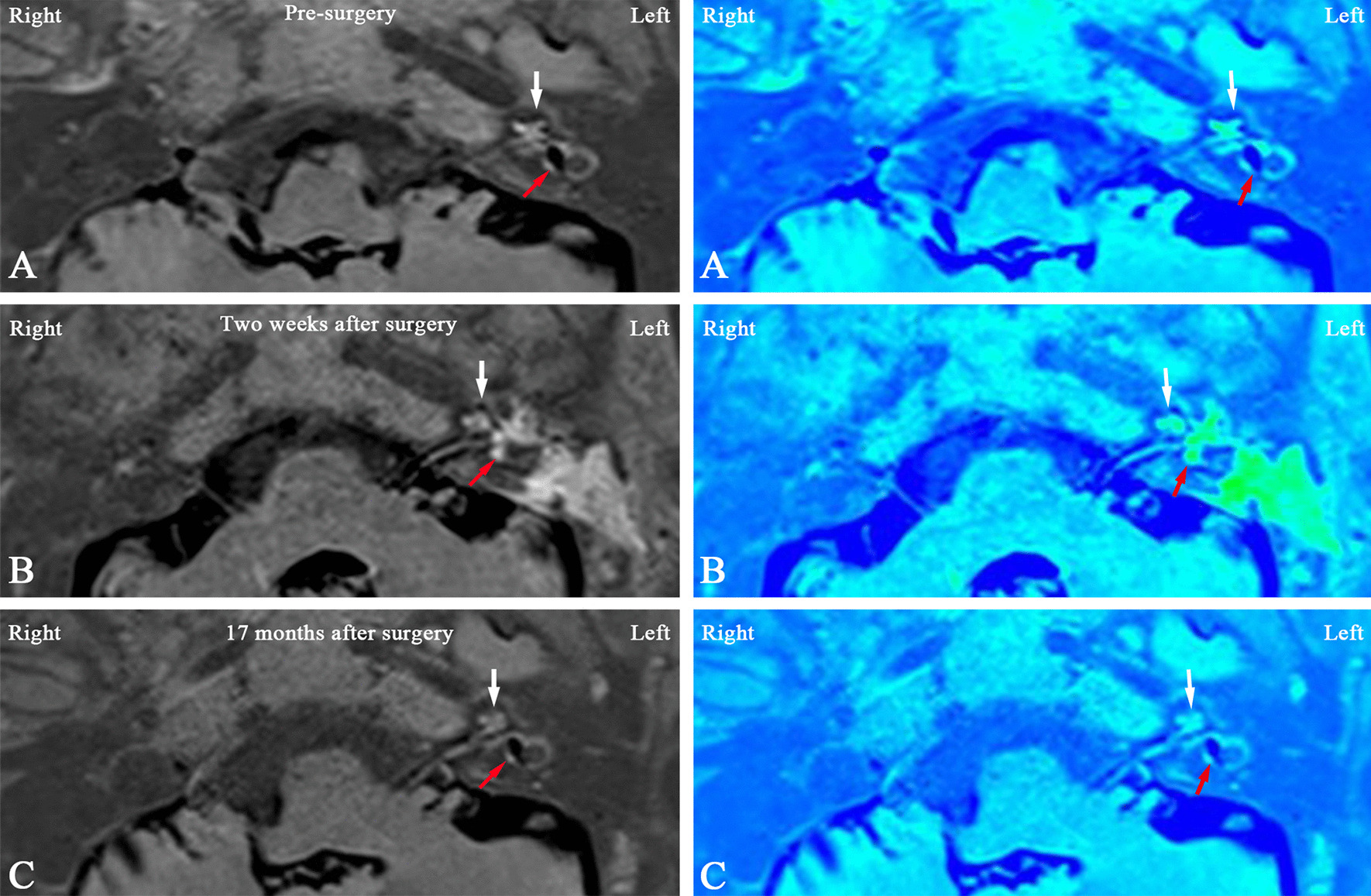
Three-dimensional real inversion recovery MRI axial scans (left column) and the corresponding MR colored image (right column) of patient no. 14 in the EDD group (Table 1) with left MD **A** prior to surgery, **B** 2 weeks after surgery, and **C** 17 months after surgery. **A** The first MRI examination, exhibiting a mild cochlear EH (white arrow) and a significant vestibular EH (red arrow) in the left ear. **B** The second MRI examination showed complete reversal of vestibular (red arrow) and cochlear EH (white arrow), in comparison with the first MRI examination in the same slice level. **C** The third MRI examination, revealing that the reversal of cochlear hydrops remained unchanged (white arrow), and there was a recurrence of vestibular hydrops (red arrow) in comparison with the first and second MRI examinations in the same slice level. *EDD* endolymphatic sac drainage, *EH* endolymphatic hydrops, *MD* Meniere’s disease, *MRI* magnetic resonance imaging

**Fig. 5:**
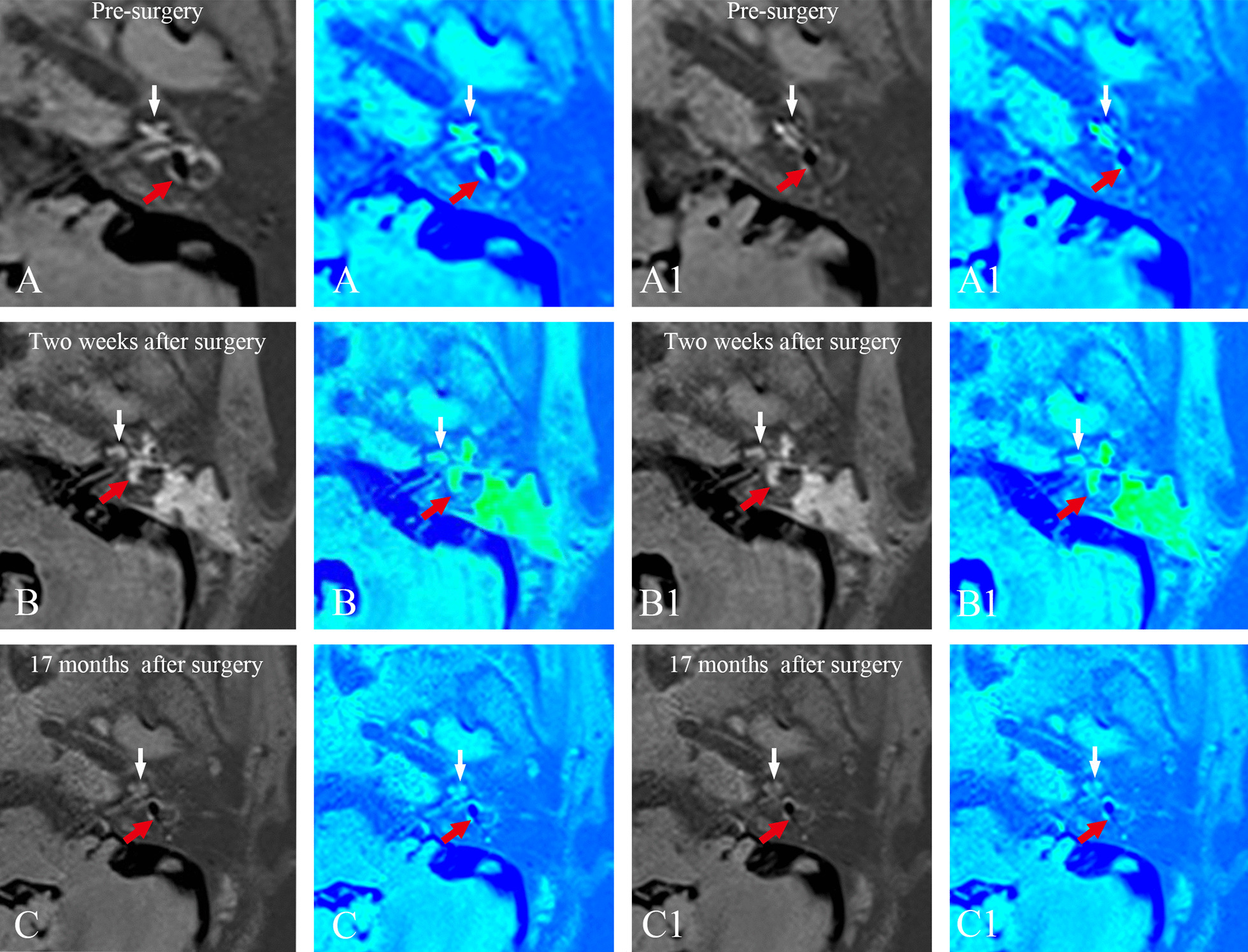
Serial MRI axial scans (left column) and the corresponding MRI colored images (right column) revealed a mild cochlear EH (white arrow) and a significant vestibular EH (red arrow) prior to surgery (**A, A1**), a complete reversal of vestibular (red arrow) and cochlear EH (white arrow) 2 weeks after surgery (**B, B1**), and the reversal of cochlear hydrops unchanged (white arrow) and a recurrence of vestibular hydrops (red arrow) 17 months after surgery (**C, C1**) in the same patient in Fig. 4. *EH* endolymphatic hydrops, *MRI* magnetic resonance imaging

In the group with endolymphatic sac drainage (patient case nos. 11–19), the second MRI examination revealed the reversal of both vestibular and cochlear hydrops in 4 patients, but no changes in EH in the remaining 5 patients. The third MRI examinations revealed that the reversal of EH in the 4 patients that were identified in the second MRI scans remained unchanged, except for one patient, for whom a recurrence of vestibular hydrops was detected. In all these 4 patients who showed a reversal of EH, the vertigo attacks were completely controlled, whereas hearing improved only in one of them, worsened in another patient, and was stabilized in the other two. One patient showed an increase in vestibular EH, accompanied by no changes in hearing and a reduction in vertigo attacks. The other 4 patients showed no changes in EH, with one patient exhibiting a worsening of their hearing, although hearing was unchanged in the remaining three, and complete control of vertigo attacks was recorded for one patient, whereas vertigo attacks were only substantially controlled in the other three. Figures 4 and 5 show the dynamics of EH in patient no. 14. The second MRI examination showed complete reversal of vestibular and cochlear EH, with a decreased EVVR from 62.56% (first MRI scan) to 12.25%, and a downgrading of the cochlear hydrops from grade I (first MRI scan) to grade N, whereas the results of the third MRI showed that the reversal of cochlear hydrops remained unchanged, although a recurrence of vestibular hydrops was found.

**Fig. 6:**
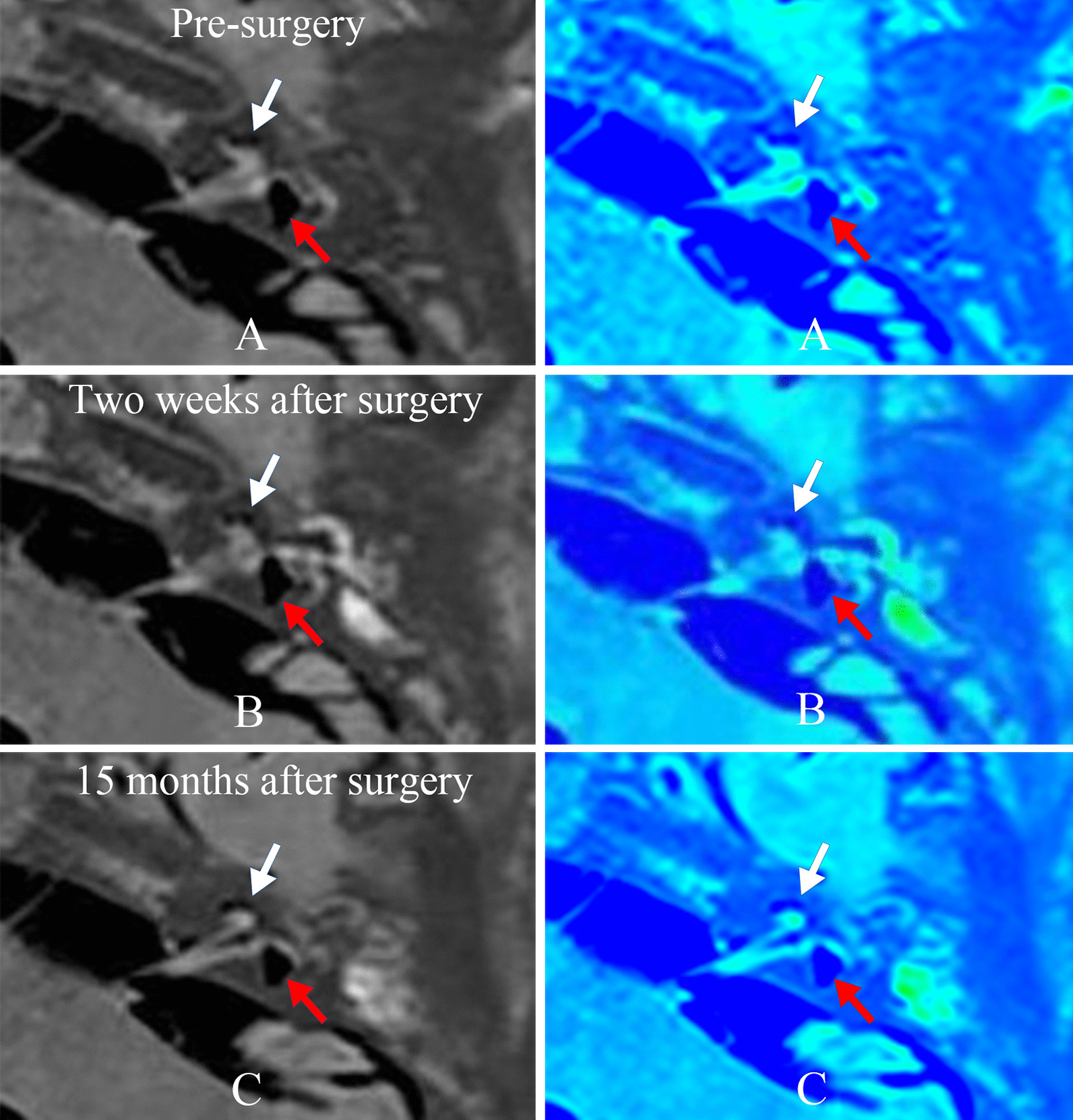
**A** Gd-MRI (left column) and the corresponding MRI colored images (right column) showed a significant cochlear (white arrow) and vestibular hydrops (red arrow) in the left ear of patient no. 23 (Table 1) before surgery. Postoperative images showed no changes of cochlear EH (white arrow) and vestibular EH (red arrow) both 2 weeks (**B**) and 15 months (**C**) following ESD surgery comparing with pre-surgery recording. *Gd-MRI* gadopentetate dimeglumine magnetic resonance imaging, *EH* endolymphatic hydrops, *ESD* endolymphatic sac decompression, *MRI* magnetic resonance imaging

In the group with endolymphatic sac decompression (patient case nos. 20–27), both the second and third MRI examinations revealed no changes in EH. Yet, the vertigo attacks was completely controlled in 2 patients and substantially controlled in 3 patients; the other 3 patients exhibited limited control of vertigo attacks in 2 and insignificant control of vertigo attacks in 1. Hearing improved in 1 patient and worsened in 1 patient. The other 6 patients showed no changes in hearing. Figure 6 shows no change of EH in patient no. 23 both 2 weeks and 15 months following surgery comparing with the pre-surgery recording.

## Discussion

In vivo visualization of hydrops with Gd-MRI is no longer limited to only showing evidence of the hydrops for the diagnosis of MD. Changes in EH may be used to objectively evaluate and differentiate the effects of various treatments for patients with MD [9, 14, 16]. In the present study, a reduction in EH was found in 3 of 10 patients receiving endolymphatic duct blackage surgery, and 4 of 9 patients receiving endolymphatic sac drainage surgery, suggesting that both endolymphatic duct blackage surgery and endolymphatic sac drainage surgery had the potential to reduce EH in certain patients with MD. However, 6 of 10 patients in the group with endolymphatic duct blackage, and 4 of 9 patients in the group with endolymphatic sac drainage did not exhibit any change in their EH, indicating that sac surgery does not always result in an improvement of EH in MD. However, both the second and third MRI examination in the group with endolymphatic sac decompression revealed no changes in EH, suggesting endolymphatic sac decompression surgery could not reduce EH in MD patients. An increased EH was identified with 1 patient who underwent endolymphatic duct blackage surgery, and 1 patient who underwent endolymphatic sac drainage surgery, and this may have been due to the natural course of MD with the progression of EH [17, 18].

Physiologically, endolymphatic duct blackage surgery is based on the hypothesis that an increased secretion outweighs a decreased absorption, resulting in increased pressure in the inner ear [7, 8]. Therefore, by blocking the endolymphatic duct, EH may be decreased due to a reduction in the volume of endolymph in the inner ear arising from the sac, as evidenced by the secretion of glycoproteins and the possible existence of hypersecretions of endolymph in the sac [19–21]. By contrast, endolymphatic sac decompression surgery and endolymphatic sac drainage surgery are based on the hypothesis that deficient absorption in the endolymphatic sac is one of the causes of EH. Although the present study showed no changes in EH in the patients following endolymphatic sac decompression surgery, yet, endolymphatic sac drainage surgery and endolymphatic duct blackage surgery were confirmed to have the potential to reduce EH in some MD patients. However, the reversal of EH achieved in certain patients with MD via the two opposing surgical approaches on the endolymphatic sac, as investigated in the present study, has demonstrated the inhomogeneity and complexity of the mechanisms underpinning the development of EH. The exact role of the endolymphatic sac in MD remains unknown. Interestingly, in the group with endolymphatic duct blackage, the EH remained unchanged 2 weeks after surgery, and the reversal of EH could only be detected in some of the patients at > 12 months following surgery, suggesting the reduction in EH was likely due to a delayed effect associated with endolymphatic duct blackage surgery that progressed over time. By contrast, in the group with endolymphatic sac drainage, the reversal of EH could be detected 2 weeks following surgery, suggesting that endolymphatic sac drainage surgery resulted in the reduction in EH very soon after surgery, which was likely due to the mechanical effect of the opened endolymphatic sac with an acute reversal of EH. Since no further patients showed any reduction in the endolymph space at > 12 months following surgery-indeed, a recurrence of vestibular hydrops in one patient was detected in comparison with the results of Gd-MRI 2 weeks following surgery-a delayed effect for reducing EH was not found in EDD group. Although a precise understanding of the physiological mechanisms underlying MD-related vertigo has yet to be elucidated, vertiginous attack is considered to result from acute development or exacerbation of EH [22], suggesting that the reduced in EH was likely to have been associated with a reduction of spells. This hypothesis seems likely to explain how a complete control of vertigo was achieved in the patients in both the group with endolymphatic duct blackage and the group with endolymphatic sac drainage who exhibited a reversal of EH in the present study. In addition, a major body of evidence already exists in support of a direct link between low-tone sensorineural hearing loss and EH in MD [1]; therefore, a reduction in EH is expected to result in an improvement in hearing. In the present study, all 3 patients in the group with endolymphatic duct blackage for whom reversal of EH was confirmed were found to have improved hearing, revealing a cause-effect relationship between EH and hearing function. However, with the 4 patients in the group with endolymphatic sac drainage for whom reversal of EH was confirmed, hearing improved in only one patient, worsened in one, and was stabilized in the other two, suggesting no correlation between the changes in hearing function and the volume of EH after sac drainage surgery, a finding that was consistent with previous reports [23–25]. However, some patients in the present study who had complete or substantial control of vertigo attacks did not exhibit changes in their hydrops following surgery, suggesting that vertigo control may be achieved even without a reduction in hydrops. The reasons for this result are unclear. The symptomatic improvement in these patients may have been a coincidence associated with symptom fluctuation of the disease or a potential placebo effect of surgery [26], and will be further studied. In fact, the mechanism of vertigo attack is not completely understood and is not explained by the state of endolymphatic hydrops alone [27].

There are some limitations in the present study. First, this study did not take into account hearing at 250 Hz and word recognition score (WRS) in all participants prior to and following surgery, which might affect the evaluation of hearing function in these patients with MD receiving surgical treatment. Second, there was a lack of pathological evidence in the endolymphatic sac to explain why the reversal of EH could be achieved via two opposing surgical approaches on the sac, which needs to be further investigated in future research.

## Conclusion

The present study has indicated that both endolymphatic duct blackage surgery and endolymphatic sac drainage surgery have the potential to reduce EH in certain patients with MD, whereas endolymphatic sac decompression surgery could not reduce EH in MD patients. In patients receiving endolymphatic duct blackage, the reversal of EH presented with complete control of vertigo and hearing improvement; whereas in patients receiving endolymphatic sac drainage, the reduction in EH presented only with complete control of vertigo, without cause-effect association with hearing function.
